# Analysis of the Na^+^/Ca^2+^ Exchanger Gene Family within the Phylum Nematoda

**DOI:** 10.1371/journal.pone.0112841

**Published:** 2014-11-14

**Authors:** Chao He, Damien M. O'Halloran

**Affiliations:** 1 Department of Biological Sciences, The George Washington University, Washington, D.C., United States of America; 2 Institute for Neuroscience, The George Washington University, Washington, D.C., United States of America; Centre National de la Recherche Scientique & University of Nice Sophia-Antipolis, France

## Abstract

Na^+^/Ca^2+^ exchangers are low affinity, high capacity transporters that rapidly transport calcium at the plasma membrane, mitochondrion, endoplasmic (and sarcoplasmic) reticulum, and the nucleus. Na^+^/Ca^2+^ exchangers are widely expressed in diverse cell types where they contribute homeostatic balance to calcium levels. In animals, Na^+^/Ca^2+^ exchangers are divided into three groups based upon stoichiometry: Na^+^/Ca^2+^ exchangers (NCX), Na^+^/Ca^2+^/K^+^ exchangers (NCKX), and Ca^2+^/Cation exchangers (CCX). In mammals there are three NCX genes, five NCKX genes and one CCX (NCLX) gene. The genome of the nematode *Caenorhabditis elegans* contains ten Na^+^/Ca^2+^ exchanger genes: three NCX; five CCX; and two NCKX genes. Here we set out to characterize structural and taxonomic specializations within the family of Na^+^/Ca^2+^ exchangers across the phylum Nematoda. In this analysis we identify Na^+^/Ca^2+^ exchanger genes from twelve species of nematodes and reconstruct their phylogenetic and evolutionary relationships. The most notable feature of the resulting phylogenies was the heterogeneous evolution observed within exchanger subtypes. Specifically, in the case of the CCX exchangers we did not detect members of this class in three Clade III nematodes. Within the *Caenorhabditis* and *Pristionchus* lineages we identify between three and five CCX representatives, whereas in other Clade V and also Clade IV nematode taxa we only observed a single CCX gene in each species, and in the Clade III nematode taxa that we sampled we identify NCX and NCKX encoding genes but no evidence of CCX representatives using our mining approach. We also provided re-annotation for predicted CCX gene structures from *Heterorhabditis bacteriophora* and *Caenorhabditis japonica* by RT-PCR and sequencing. Together, these findings reveal a complex picture of Na^+^/Ca^2+^ transporters in nematodes that suggest an incongruent evolutionary history of proteins that provide central control of calcium dynamics.

## Introduction

Na^+^/Ca^2+^ exchangers are a family of proteins that provide homeostatic balance to the cell's calcium concentration. Na^+^/Ca^2+^ exchangers are divided into three groups in animals based upon their stoichiometry: Na^+^/Ca^2+^ exchangers (NCX) which exchange sodium for calcium, Na^+^/Ca^2+^/K^+^ exchangers (NCKX) which exchange sodium for potassium and calcium, and Ca^2+^/Cation exchangers (CCX; also called NCLX) which exchange sodium or lithium for calcium [1]–[3]. Na^+^/Ca^2+^ exchangers have been shown to regulate calcium exchange at the cell membrane, endoplasmic reticulum, mitochondrion, and at the nucleus [4]–[6]. NCX, NCKX, and CCX exchangers are low affinity/high capacity ion transporters and can rapidly expel (forward mode) or introduce (reverse mode) calcium ions to the cell or organelle. All Na^+^/Ca^2+^ exchangers contain a tandemly repeated protein motif, the alpha repeat, which invariably occurs in two blocks of five transmembrane domains separated by a cytoplasmic loop; with some variations, the residues of the alpha repeat are conserved among all three classes of Na^+^/Ca^2+^ exchanger and in all organisms; and where perturbed experimentally, these residues have been shown to be crucial for exchanger function [1]. NCX proteins are comprised of ten transmembrane domains [7], [8], including an intracellular loop between TM5 and TM6 that contains the calcium binding domain 1 (CBD1) and calcium binding domain 2 (CBD2) that represent regulatory domains required for intracellular ion sensing [9]–[11]. At the primary sequence level, these tandem CBD1 and CBD2 domains both correspond with the CalX-beta motif, which is found tandemly repeated in essentially all NCX-class exchangers examined, and which is therefore a diagnostic marker distinguishing NCX-class from CCX-class exchangers [11]. The NCX and NCKX exchangers share sequence similarity in the transport α-repeat domains: G(S/G)SAPE within the α1 repeat, and GTS(I/V)PD within the α2 repeat. The CCX exchanger has a unique conserved sequence within the α-repeats: GNG(A/S)PD in α1 and (G/S)(N/D)SxGD in α2. Three NCX genes, five NCKX genes, and one CCX gene have been cloned and identified in mammals. Mammalian NCXs (NCX1-3) are highly expressed in cardiac muscle, skeletal muscle, and the central nervous system [5], [12], [13]. Mammalian NCKX1-5 are widely expressed in various cells including rod and cone photoreceptor cells, retinal ganglion cells, platelets, vascular smooth muscles, uterus, brain tissue, intestine, lungs, thymus, and epidermal cells [14]–[17]. The mammalian CCX exchanger NCLX (also termed NCKX6) is expressed in all tissues examined including the brain, thymus, heart, skeletal muscles, lungs, kidneys, intestines and testes and has been shown to localize to mitochondria [18]–[20]. Functionally, Na^+^/Ca^2+^ exchangers contribute to the normal physiology of a wide variety of cells and tissues. Na^+^/Ca^2^ based exchange is considered the principal method of Ca^2+^ removal during heartbeat [21], and within the hippocampus NCX2 and NCX3 has been shown to contribute to plasticity and learning behavior [22], [23].

In *Caenorhabditis elegans*, a total of ten Na^+^/Ca^2+^ exchanger genes have been identified in the *C. elegans* genome (designated *ncx-1* to *ncx-10*) [1], [24], [25]. There are three NCX genes (*ncx-1, ncx-2* and *ncx-3*); *ncx-4* and *ncx-5* encode for proteins that belong to the NCKX branch; and *ncx-6* – *ncx-10* encode for CCX representatives in *C. elegans*. Here we set out to characterize structural and taxonomic specializations within the family of Na^+^/Ca^2+^ exchangers across the phylum Nematoda. We sourced members of the Na^+^/Ca^2+^ exchanger family in the following twelve nematode species (Clade designations described by Blaxter et al. [26]): Clade IV - *Strongyloides ratti*, Clade V *- Haemonchus contortus, Heterorhabditis bacteriophora, Caenorhabditis elegans, Caenorhabditis brenneri, Caenorhabditis japonica, Caenorhabditis briggsae, Caenorhabditis remanei, Pristionchus pacificus*, Clade III - *Brugia malayi, Loa loa, and Ascaris suum*. From these sequences we then reconstructed the phylogenetic relationship for NCX, NCKX, and NCLX across all twelve species and investigated rates of selection for each transporter type. Na^+^/Ca^2+^ exchangers are highly conserved across mammalian taxa at the protein and syntenic levels [1], [27], and from our analysis we observed an unexpected level of heterogeneity in copy number within nematodes, in particular within the CCX subtype where we detected several putative examples of gene gain and/or loss. We detected between three and five CCX members across *Caenorhabditis* and *P. pacificus* species, and single CCX proteins for *H. contortus*, *H. bacteriophora*, and *S. ratti*, and did not detect any CCX members within *B. malayi, L. loa, or A. suum*. We also provided re-annotation for gene structure predictions for CCX members within *C. japonica* and *H. bacteriophora* by RT-PCR and sequencing.

## Materials and Methods

### Sequences

**Figure 1:**
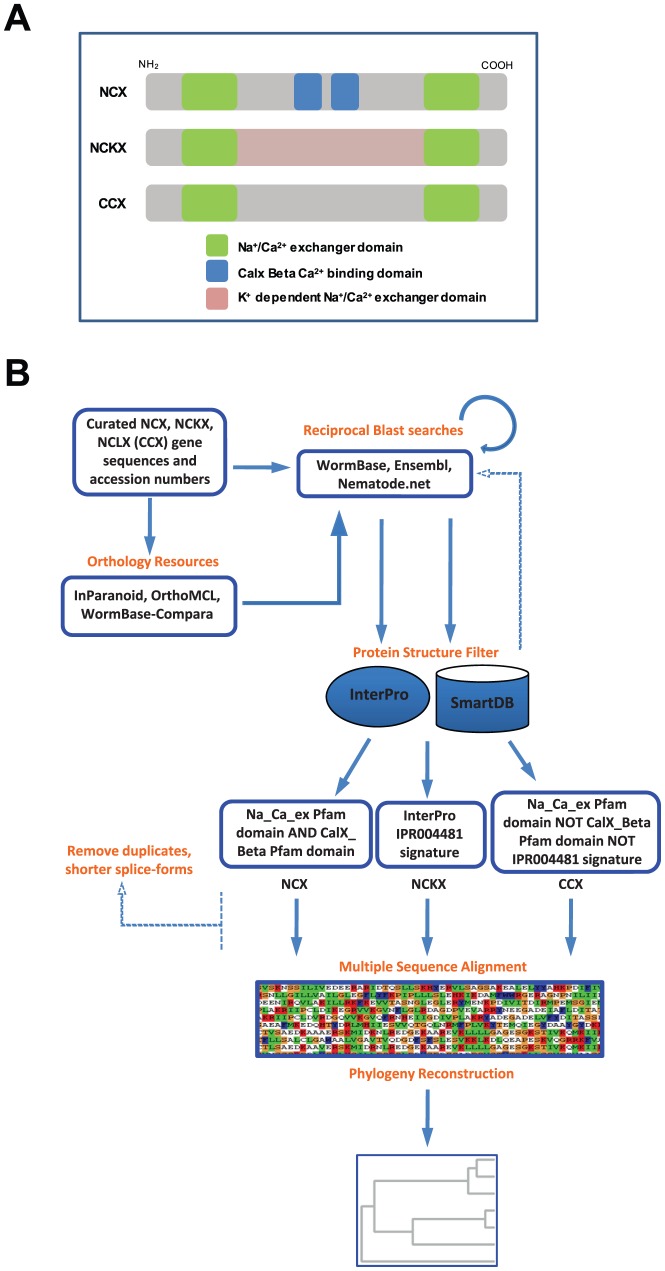
Structures within NCX, NCKX, and CCX proteins, and an overview of the pipeline used to detect orthologs of these proteins in twelve species of nematodes. (**A**) Cartoon depicting structures within the NCX, NCKX, and CCX (NCLX) proteins. (**B**) Overview of a pipeline used to detect orthologous sodium calcium exchanger genes in twelve different species of nematodes.

The genomes of the nematodes sampled (Strongyloides ratti, Haemonchus contortus, Heterorhabditis bacteriophora, Caenorhabditis elegans, Caenorhabditis brenneri, Caenorhabditis japonica, Caenorhabditis briggsae, Caenorhabditis remanei, Pristionchus pacificus, Brugia malayi, Loa loa, and Ascaris suum) were searched for NCX, NCKX and CCX protein sequences with bidirectional BlastX and BlastP searches using WormBase ver.WS243 [28], Ensembl [29], Nematode.net [30], InParanoid [31]; and OrthoMCL [32] using curated NCX, NCKX, and CCX sequences from C. elegans, Drosophila melanogaster, and mammals as starting material. Specific structural signatures unique to each sodium calcium exchanger subtype were used to parse matches (Figure 1A), which were filtered through Interpro [33] and SMARTDB [34] to organize hits into either: Sodium Calcium Exchangers (NCX), Potassium dependent Sodium Calcium Exchangers (NCKX), or Sodium Calcium Lithium Exchanger (NCLX [aka CCX]) subtypes (Figure 1B).

### Sequence Analysis

Proteins were aligned using the multiple sequence alignment software MUSCLE v3.8.31 [35], and gaps were systematically stripped after alignment. The appropriate model was selected using Prottest v3 [36], [37] and found to be LG+I+G+F for NCX phylogeny with gamma distribution parameter  = 1.06 and four substitution rate categories and proportion of invariant sites  = 0.05, WAG+G+F for NCKX phylogeny with gamma distribution parameter  = 0.8 and four substitution rate categories, and WAG+I+G+F for CCX phylogeny with gamma distribution parameter  = 1.03 and four substitution rate categories and proportion of invariant sites  = 0.04. Phylogenetic relationships were inferred by reconstructing trees by Maximum Likelihood using the PhyML command-line application [38] as described previously [39]. Signatures of selection were detected using the single-likelihood ancestor counting (SLAC), random effects likelihood (REL) [40], and mixed effects model of evolution (MEME) [41] methods implemented in the HyPhy package [42], [43]. DNA sequences were tested for best fit models using jModelTest [44] and recoded into codon based alignments using Pal2Nal [45]. High resolution images of alignments were obtained using Geneious [46]. Pairwise patterns of molecular diversity (π) for NCX, NCKX, and CCX exchangers between *C. elegans* and *C. briggsae* were calculated using DnaSP ver.5 [47]. NCX protein structure was predicted using Phyre [48] which incorporated the resolved NCX structure from Liao et al (PDB ID 3V5U) [8], and visualized using RasMol [49]. The alignments from our sequence analyses were used to generate a position specific weight matrix (PSWM) based upon the divergent alpha repeat structures detected across the twelve nematode species we analyzed. Using this PSWM we developed a web-based tool called ‘N(em)CX’ that searches for divergent NCX-like proteins. The server side script was written in Perl using the CGI.pm Perl module to generate output html. N(em)CX is available here: http://ohalloranlab.net/NemCX.html

### Strains and maintenance

*Caenorhabditis japonica* strain DF5081 was maintained at 20°C by mating males and females on NGM plates seeded with *E. coli* strain OP50 [50], [51]. *Heterorhabditis bacteriophora* strain TTO1 animals (kindly provided by John Hawdon) were cultured *in vivo* at 25°C in *Galleria mellonella* (wax moth) larvae using standard protocols [52]. Parasitized larvae were placed on water traps [53] to collect the infective-stage nematodes. The water traps were constructed and nematodes harvested as described previously [54], [55]. Parasitic juvenile stages of *H. bacteriophora* were harvested by obtaining *G. mellonella* cadavers 6 to 8 days post-infection and cutting them open in a Petri dish containing M9 buffer, and the emerging nematodes were washed twice with distilled water. Female adult nematodes were obtained by dissecting *G. mellonella* cadavers 5 to 7 days post-infection in a Petri dish containing M9 buffer and females were picked using an aspirator.

### DNA, RNA isolation, and RT-PCR

Genomic DNA was isolated by harvesting animals and collecting in a 1.5 ml tube. 200 µl of Lysis buffer (60 g/ml proteinase K, 10 mM Tris-Cl, pH 8.3, 50 mM KCl, 2.5 mM MgCl_2_, 0.45% IGEPAL, 0.45% Tween-20, 0.01% gelatin) was added to the tube and then frozen at −80°C for 10 mins followed by incubation at 60°C for 1 hr followed by 95°C for 15 mins. In the case of *H. bacteriophora*, animals were crushed into a fine powder using a pestle and mortar. Tubes were then centrifuged at 13,000 rpm for 1 min and ∼50 µl gDNA supernatant isolated for PCR. For *H. bacteriophora*, an extra step of phenol-chloroform purification was performed by adding a 1∶1 volume to gDNA supernatant. Total RNA was isolated from mixed stage animals using 1 ml Trizol Reagent (Invitrogen, Life Technologies, Carlsbad, CA). A 20 G syringe (Becton-Dickinson 3 ml syringe) was used to break down material and 200 µl of chloroform was added to isolate RNA from the sample. Tubes were vigorously shaken followed by centrifuging at 13,000 rpm for 10 min. The clear supernatant mixed with 1 volume of 70% EtOH was then cleaned using an RNA mini kit (Invitrogen, Life Technologies, Carlsbad, CA). The concentration and purity of the RNA samples were determined using spectrophotometry and ethidium bromide visualization of intact 18S and 28S RNA bands after agarose gel electrophoresis. Total RNA was treated with DNase (Thermo Scientific, Waltham, MA) by incubating at 37°C for 30 min followed by 65°C for 10 min, and reverse transcribed (RT) with MMLV reverse transcriptase (50 U, USB, Affymetrix, Santa Clara, CA) in 5× RT polymerase chain reaction (PCR) buffer (500 mM KCl and 100 mM Tris-HCl, pH 8.3, 7.5 mM MgCl_2_), 4 U RNase inhibitor, 10 mM each of the dNTPs. The final PCR products were electrophoresed on 1.5% agarose gels. Primer pairs used for *Caenorhabditis japonica* were CJ-F TACGTGAGCCATGGACATCACA and CJ-R TCGATACGTTGGATTGAGAATC, and primer pairs used for *Heterorhabditis bacteriophora* were: Hba-F TGCTCTACTCCTTGCTTCGTGCCCCGTA, Hba-R GGGAGTTACATTCATGGCATTTGGCAATGG using the following cycling conditions for *C. japonica*: 95°C for 3 mins, 95°C for 30 sec, 56°C for 30 sec, and 72°C extension for 3 mins 30 sec for gDNA and 1 min for cDNA; and the following cycling conditions for *H. bacteriophora*: 95°C for 3 mins, 95°C for 30 sec, 56°C for 30 sec, and 72°C extension for 1 min 40 sec for gDNA and 1 min for cDNA.

## Results

### Detecting Orthologous Sodium Calcium Exchangers

Na^+^/Ca^2+^ exchangers from *Caenorhabditis elegans* (NCX-1 to NCX-10) and mammals (NCX1-3, NCKX1-5, NCLX) were used to search various databases and resources: WormBase ver. WS243 [28], Nematode.net [30], Ensembl [29], InParanoid [31], and OrthoMCL [32] to obtain orthologs from the following nematodes: *Strongyloides ratti, Haemonchus contortus, Heterorhabditis bacteriophora, Caenorhabditis elegans, Caenorhabditis brenneri, Caenorhabditis japonica, Caenorhabditis briggsae, Caenorhabditis remanei, Pristionchus pacificus, Brugia malayi, Loa loa, and Ascaris suum*. All hits were filtered through InterPro [33] and SmartDB [34] to separate into NCX, NCKX or CCX proteins based upon unique structures (Figure 1A) within each subtype of exchanger (Figure 1B). Only NCKX members harbor a potassium dependent exchanger domain (InterPro IPR004481 signature), whereas NCX proteins contain exchanger domains (Na_Ca_ex Pfam domain) and CalX beta domains (CalX_ Beta Pfam domain), and finally CCX members do not contain the CalX beta domains but do contain the exchanger domains (Na_Ca_ex Pfam domain). In each case we used validated NCX, NCKX, and NCLX (CCX) proteins from mammals as positive controls to ensure the pipeline parsed the hits appropriately.

### NCX Phylogeny

**Figure 2:**
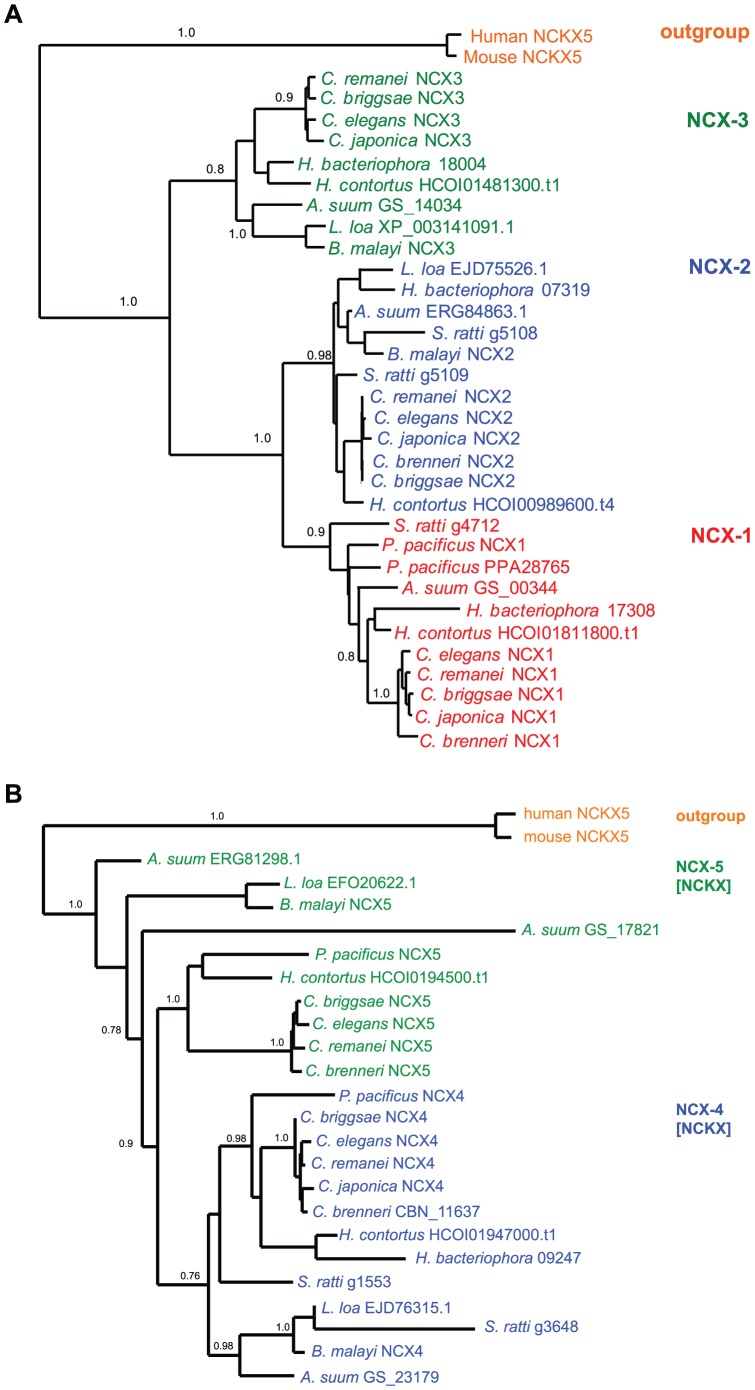
Phylogenetic analysis of NCX and NCKX exchangers in various nematodes. (**A**) Phylogenetic analysis of NCX type exchangers from *Strongyloides ratti, Haemonchus contortus, Heterorhabditis bacteriophora, Caenorhabditis elegans, Caenorhabditis brenneri, Caenorhabditis japonica, Caenorhabditis briggsae, Caenorhabditis remanei, Pristionchus pacificus, Brugia malayi, Loa loa*, and *Ascaris suum*. Inferred phylogeny was constructed using PhyML [38] and derived from amino acid alignments using MUSCLE [35]. The NCKX5 exchanger from human and mouse was used as an outgroup. (**B**) Phylogenetic analysis of NCKX type exchangers from *S. ratti, H. contortus, H. bacteriophora, C. elegans, C. brenneri, C. japonica, C. briggsae, C. remanei, P. pacificus, B. malayi, L. loa*, and *A. suum*. Inferred phylogeny was constructed using PhyML [38] using the model WAG+G+F determined from Prottest [36] and derived from amino acid alignments using MUSCLE [35]. The NCKX type exchangers from human and mouse served as an outgroup.

**Figure 3:**
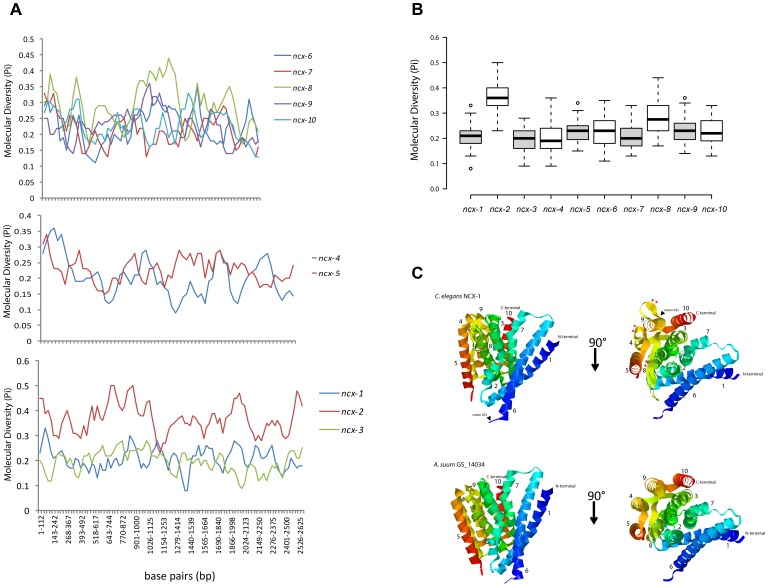
Sequence and structural analysis of nematode exchangers. (**A**) Sliding window analysis of nucleotide diversity using DnaSP version 5 [47] with 100 bp windows and 25 bp steps for CCX (upper graph), NCKX (middle graph), and NCX exchanger (lower graph) gene pairs between *C. elegans* and *C. briggsae*. (**B**) Box plot analysis of molecular diversity for each *ncx* gene (*ncx-1* to *ncx-10*) between *C. elegans* and *C. briggsae*. Whiskers extend to data points that are less than 1.5 × interquartile range away from 1st/3rd quartile; center lines show the medians and outliers are shown as dots. (**C**) Ribbon model of NCX-1 from *C. elegans* and an NCX-3 ortholog from *A. suum* (GS_14034). N and C termini are indicated, and red arrowheads refer to structural differences between each NCX. Numbers refer to the transmembrane (TM) domains. Extracellular side is up in left views. The position of two candidate sites in NCX-1 undergoing episodic diversifying selection are indicted - codon 455 and codon 925. In each case the right view is rotated by 90°. Structural predictions were made using Phyre [48] and visualized using RasMol [49]. In each case the single highest scoring modelling template was the resolved NCX (NCX_Mj) structure (PDB: 3V5U) from Liao et al. [8].

The NCX Na^+^/Ca^2+^ exchangers were mostly conserved across all species examined (Figure 2A). NCX-1 and NCX-2 are most closely related in each case with NCX-3 representing a more diversified clade. The alpha repeat domains were highly conserved and adopted the consensus GSSAPE for the α1 repeat and GTS(I/V/L)PD for the α2 repeat. Together, these repeats comprise four transmembrane domains (TM2, TM3, TM7, TM8) that form a diamond shaped transport vestibule [8]. We identified three NCX genes for each *Caenorhabditis* species that we examined except in the case of *C. brenneri* where we only identified two NCX genes. For each of the NCX clusters, the *Caenorhabditis* orthologs grouped together (Figure 2A). For the NCX-1 and NCX-3 groups, the *H. bacteriophora* and *H. contortus* orthologs grouped together, and for NCX-3, the *B. malayi*, *L. loa*, and *A. suum* orthologs grouped close together (Figure 2A). Outside of the *Caenorhabditis* genus, we identified three NCX members for each species except *P. pacificus*, *B. malayi* and *L. loa* which each were assigned two NCX genes. In the case of *P. pacificus*, each NCX gene grouped into the NCX-1 cluster. Similarly, for *S. ratti*, two of its three NCX genes grouped into the NCX-2 cluster. In cases where multiple hits were detected within each cluster, we re-examined the alignment to ensure alternative splicing was not a misleading factor. We examined selection across the NCX *Caenorhabditis* taxa using MEME [41] and found a global dN/dS value  = 0.130, we also used SLAC [40] and found a global dN/dS value  = 0.138955 (*p*<0.01). We also conducted a sliding window analysis of nucleotide diversity between *C. elegans* and *C. briggsae* NCX genes using DnaSP ver. 5 [47], and observed similar polymorphic patterns across the *ncx-1* and *ncx-3* sequences (average π score  = 0.2075 for *ncx-1*, and 0.1955 for *ncx-3*), and slightly elevated levels of diversity for *ncx-2* (average π score  = 0.369) (Figure 3A–3B). However, elevated nucleotide diversity for *ncx-2* does not hold for other *Caenorhabditis* pairs: for example using *C. elegans* and *C. japonica* the average *ncx-2* π score  = 0.173 (sampling variance  = 0.007), and using *C. elegans* and *C. remanei* the average *ncx-2* π score  = 0.11 (sampling variance  = 0.003). Next, we tested specific sites for positive selection using REL [40] within the *Caenorhabditis* taxa, and from this analysis we did not detect any sites undergoing positive selection. We also tested for episodic diversifying selection using MEME and detected two significant (*p*<0.01) sites: codon 455, which is positioned close to the second calcium binding domain (CBD2) between TM5 and TM6, which detects local intracellular calcium levels, and codon 925 which is located after TM9 in the intracellular loop that connects with TM10 (see Figure 3C). The crystal structure for the NCX from *Methanococcus jannaschii* (NCX_Mj) has been resolved [8], and using this structure we examined structural diversity across NCX proteins in nematodes. The NCX-1 and NCX-3 clusters are the most divergent amongst the three NCX clusters (Figure 2A), and so to examine structural differences between diverse NCX proteins in nematodes we selected a representative from the NCX-1 cluster (*C. elegans* NCX-1) and a representative from the NCX-3 cluster (*A. suum* GS_14034) for *in silico* structural analysis. For NCX-1 from *C. elegans* 32% of the residues (285 residues in total) were modelled at 100% confidence and yielded 33% alpha helical and 20% beta strand structures. The *A. suum* GS_14034 NCX was modelled at 100% confidence for 36% of the sequence (289 residues) including 36% alpha helical and 22% beta strand structures. In each case the single highest scoring modelling template was the resolved NCX_Mj structure (PDB: 3V5U) from Liao et al. [8]. In the case of NCX-1 we observed a longer beta strand structure connecting TM8 and TM9 from residues 801–817 than is predicted for *A. suum* GS_14034 (see red arrowheads, Figure 3C), and similarly another lengthy beta structure is predicted for NCX-1 connecting TM4 to TM5, and also a shorter alpha helical structure within TM4 of NCX-1 (red arrowheads, Figure 3C), that is not predicted for *A. suum* GS_14034 (Figure 3C). In the case of *A. suum* GS_14034, TM4 is composed of consistent alpha helical structure from residues 165–185, and the linker connecting TM8 and TM9 is significantly shorter in *A. suum* GS_14034 from residues 742–748 (Figure 3C).

### NCKX Phylogeny

Members of the NCKX family exhibited much diversity at the protein level and broadly assembled into representatives of NCX-4 and NCX-5 clusters. In each cluster, the *Caenorhabditis* species grouped together although we did not detect an NCX-5 member for *C. japonica* (Figure 2B). In all other species we detected two NCKX genes except for *H. bacteriophora* for which we only detected one NCKX gene and *A. suum* for which we detected three NCKX genes. In the NCX-4 cluster *H. contortus* and *H. bacteriophora* orthologs grouped together. Within the NCX-5 cluster we observed more diversity and longer branch lengths, especially true in the case of *A. suum* GS_17821 which only shares 34.9% percent identity with its nearest neighbor, *B. malayi* NCX-5. In almost all cases the highly conserved aspartic acid residue within the α2 repeat domain that confers potassium dependence [56] was present with the exception of the following proteins: *S.ratti-*g3648, *A.suum-*ERG81298.1, and *H.bacteriophora_*09247 - each of these proteins contained the conserved α1 repeat domain but were atypical for the α2 repeat domain. We also examined selection across the NCKX *Caenorhabditis* genus and found a global dN/dS value using SLAC of 0.118693 (*p*<0.01), and a global dN/dS value of 0.115 using MEME (*p*<0.01). A sliding window of nucleotide diversity was generated for each NCKX gene pair between *C. elegans* and *C. briggsae* and revealed similar patterns of DNA polymorphisms across each exchanger (average π value for *ncx-4*  = 0.203, and average π value for *ncx-5*  = 0.22) (Figure 3A–3B). Next, we tested for site specific evidence of positive selection using REL, and found evidence for one site undergoing positive selection: codon 9, which is located prior to the first predicted TM segment. Finally, we tested for specific branches undergoing episodic diversification using MEME but did not detect any evidence of episodic diversification (*p*<0.01).

### CCX (NCLX) Phylogeny

**Figure 4:**
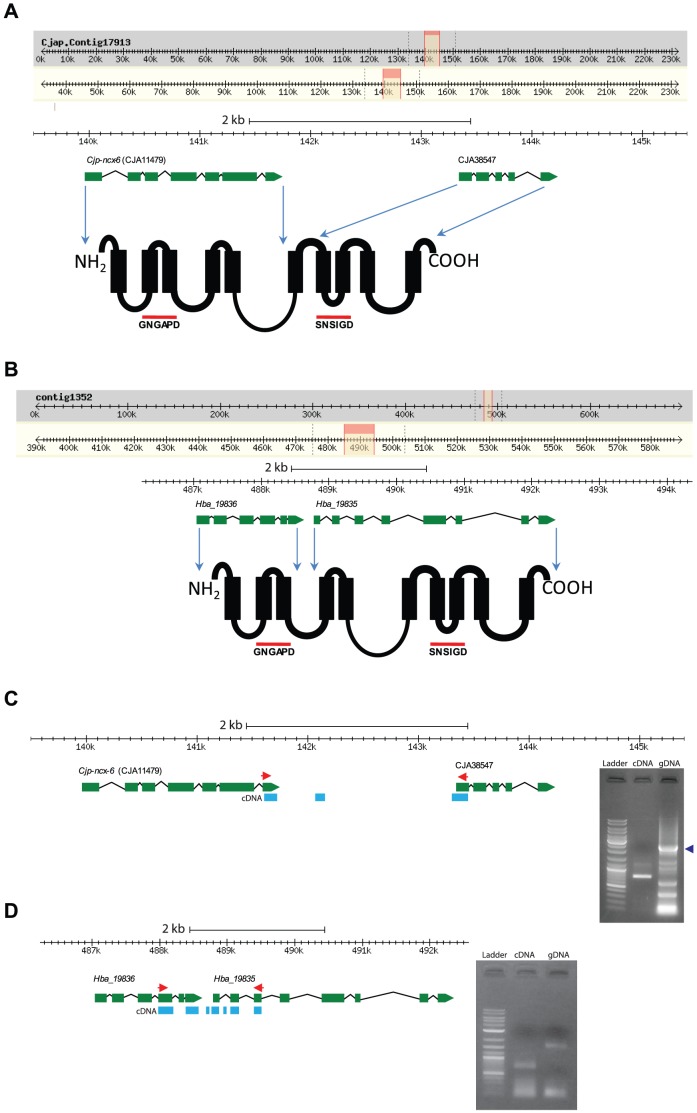
Predicted gene structure of CCX proteins from *Caenorhabditis japonica* and *Heterorhabditis bacteriophora*. (**A**) Predicted gene structures from *Cja11479* and *Cja38547* on contig 17913 from *Caenorhabditis japonica*. Translation of each predicted gene produces approximately half an NCLX-like protein containing the α1 repeat sequence GNGAPD and the α2 domain sequence SNSIGD. Blue arrows indicate the approximate position of the predicted coding sequence mapped to the translation. (**B**) Predicted gene structure from *Hba_19835* and *Hba_19836* on contig 1352 from *Heterorhabditis bacteriophora*. Translations generate approximately half an NCLX-like protein containing the α1 repeat sequence GNGAPD and the α2 domain sequence SNSIGD. Blue arrows indicate the approximate position of the predicted coding sequence mapped to the protein translation. (**C**) PCR Primers were designed that spanned the final exon of the upstream predicted gene *Cja11479* and the first exon of the downstream predicted gene *Cja38547*. Using genomic DNA as template for PCR we observed a band at 2443 bp (third lane in gel inset), and using reverse transcribed RNA as template for PCR we observed a band at 813 bp (second lane in gel inset). Primers are indicated by red arrowheads, and resulting cDNA sequence mapped to gene structure is indicated by the blue rectangles. First lane in the gel electrophoresis image is a GenRuler DNA ladder mix (Thermo Scientific - SM0334). Purple arrowhead at inset indicates the position of the gDNA band corresponding to the 2443 bp fragment. (**D**) Primers were designed that spanned the third to last exon of the upstream predicted gene *Hba_19836* and the third exon of the downstream predicted gene *Hba_19835*. Using genomic DNA as template for PCR we observed a band at 1623 bp (third lane in gel inset), and using reverse transcribed RNA as template for PCR we observed a band at 674 bp (second lane in gel inset). Primers are indicated by red arrowheads, and resulting cDNA sequence mapped to the gene structure is denoted by blue rectangles. First lane in the gel electrophoresis inset image is a GenRuler DNA ladder mix (Thermo Scientific - SM0334).

In analyzing the CCX group, we noted that for *C. japonica* two tandem predicted genes, *Cja11479* and *Cja38547*, were annotated as separate protein-coding genes, and at the translated protein level we found that each gene was predicted to encode only one half of a single CCX protein (Figure 4A). We observed a similar scenario with the *H. bacteriophora* tandem predicted genes, *Hba_19835* and *Hba_19836*, which are annotated as two separate protein coding genes, and from our analysis we found these separate predicted genes would encode one single CCX protein (Figure 4B). Interestingly, each of these predicted genes are annotated as separate genes on account of the stop codon at the end of the final exon of the upstream gene prediction in each case. Na^+^/Ca^2+^ exchangers have been shown to exhibit many alternatively spliced isoforms [57], [58], however, in these two cases considering the size of the encoded protein predicted by the shorter isoform, which would only encode a single α-repeat domain and lack critical structures necessary for sodium calcium exchange, it seems unlikely that such an isoform would be generated. To investigate this further, we examined by RT-PCR whether a single mRNA could be detected bridging both predicted genes in each case. We designed primers that spanned the final exon of the upstream gene prediction and the first exon of the downstream predicted gene in the case of *C. japonica*, and the third to last exon of the upstream gene prediction and third exon of the downstream predicted gene in the case of *H. bacteriophora* (see red arrowheads in Figure 4C–4D). Using genomic DNA as template we observed a band at 2443 bp in the case of *C. japonica*, and using reverse transcribed RNA as template we observed a cDNA band at 813 bp (see inset of gel in Figure 4C). This suggests that together these predicted genes likely produce an individual mRNA. We sequenced the RT-PCR product and found that the cDNA sequence ended 5 bp prior to the currently annotated stop codon in the final exon of *Cja11479*, and then continued in what is currently annotated as noncoding sequence for 125 bp, and then once again continued 29 bp upstream of the currently annotated start codon of the *Cja38547* gene prediction (Figure 4C, blue rectangles indicate cDNA). BlastP interrogation of the *C. elegans* genome using the protein translation from this cDNA sequence provides a top match with the *C. elegans* NCX-6 predicted protein, which includes our cDNA sequence that covers what is currently annotated as non-coding sequence between each predicted gene. This suggests misannotation in the current gene structure prediction at this locus for *C. japonica*. We adopted the same approach to investigate the *H. bacteriophora* predicted genes, *Hba_19836* and *Hba_19835*, and similarly found that a single transcript could be detected that bridges both predicted genes, suggesting again the possibility of misannotation in the current gene structure prediction at this locus (Figure 4D). We observed a band at 1623 bp using gDNA as template and a band at 674 bp using cDNA as template. We sequenced this RT-PCR cDNA product and found that the cDNA sequence ended prior to the currently annotated stop codon in the final exon of *Hba_19836*, and then continued in what is currently annotated as non-coding sequence between both predicted genes, and then continued 5 bp upstream of the currently annotated start codon of the *Hba_19835* gene (Figure 4D, blue rectangles indicate cDNA). Taken together, this also suggests misannotation in the current gene structure predictions at this locus for *H. bacteriophora*. Therefore, in the analysis that follows on the CCX exchanger phylogeny we used the translation from concatenated *Cja11479* and *Cja38547* gene predictions in the case of *C. japonica* and the translation from concatenated *H. bacteriophora* predicted genes *Hba_19835* and *Hba_19836* for our phylogenetic analyses. Our cDNA sequences for *C. japonica* and *H. bacteriophora* were deposited at NCBI's GenBank (accession number KJ873055 for *C. japonica* and KM009146 for *H. bacteriophora*).

**Figure 5:**
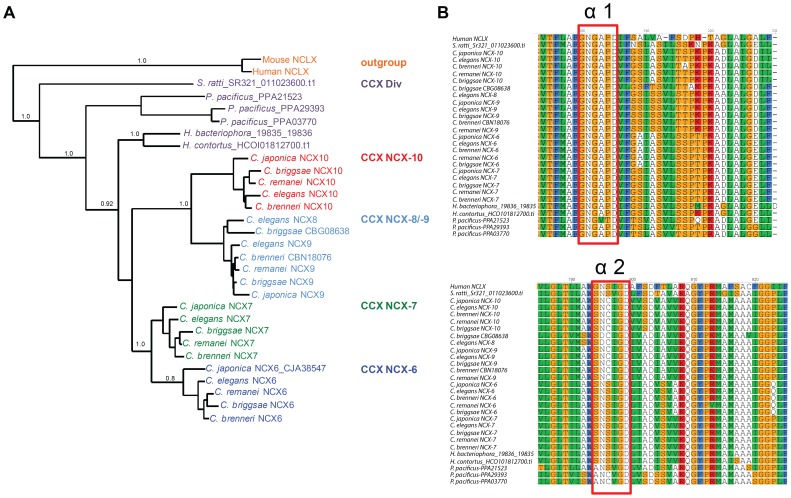
Phylogenetic analysis of NCLX (CCX) exchangers from various nematodes. (**A**) Phylogenetic analysis of NCLX type exchangers from *Strongyloides ratti, Haemonchus contortus, Heterorhabditis bacteriophora, Caenorhabditis elegans, Caenorhabditis brenneri, Caenorhabditis japonica, Caenorhabditis briggsae, Caenorhabditis remanei*, and *Pristionchus pacificus*. Inferred phylogeny was constructed using PhyML [38] using the model WAG+I+G+F determined from Prottest [36] and derived from amino acid alignments using MUSCLE [35]. ‘CCX div’ denotes a divergent CCX cluster that does not group with the *Caenorhabditis* CCX protein clusters (i.e. NCX-6 to NCX-10). The NCLX exchanger from human and mouse were used as an outgroup. (**B**) Alignment of α repeat domains within CCX (NCLX) proteins from various nematodes and also human NCLX. Alignments were generated using MUSCLE [35] of NCLX type exchangers from Human, *S. ratti, H. contortus, H. bacteriophora, C. elegans, C. brenneri, C. japonica, C. briggsae, C. remanei*, and *P. pacificus*.

The CCX nematode phylogeny revealed the most unexpected reconstruction, most notably is the gene expansion specific to the *Caenorhabditis* genus (Figure 5A). Within each *Caenorhabditis* species that we examined we detected between four and five CCX genes. We detected three CCX genes for *P. pacificus*, and all other species examined had either a single CCX member or no CCX representative. The CCX encoding genes from *H. contortus*, *H. bacteriophora*, *P. pacificus* and *S. ratti* were more divergent (‘CCX div’, Figure 5A) than those observed for *Caenorhabditis* species. Although the CCX group exhibited much diversity at the nucleotide level (Figure 3A and 3B), at the structural level this group is highly conserved as evident in the alignment of the α1 and α2 repeat domains alongside the human NCLX (Figure 5B), which comprise the GNGAPD motif for α1 and (A/S)N(S/C)(V/I)GD for the α2 repeat domain. We also examined selection across the CCX *Caenorhabditis* taxa and found a global dN/dS value  = 0.1629 using SLAC (*p*<0.01), and a global dN/dS value  = 0.1550 using MEME (*p*<0.01). We examined nucleotide diversity for each CCX gene and observed unique patterns of diversity (Figure 3A) but similar overall rates of variation (Figure 3B) for each CCX gene with the exception of *ncx-8*, which exhibited elevated levels of diversity compared with the other CCX exchangers, particularly within the region between each transport domain (Figure 3A). We identified two candidate sites undergoing positive selection: codon 17 which is positioned in the extracellular N terminal before the first TM segment, and codon 520 which is located in a large predicted intracellular loop prior to the second α repeat domain. We also implemented MEME to search for episodic diversification and found one significant (*p*<0.01) example at codon 754 of *C. briggsae* NCX-7 in close proximity to the second transport repeat domain.

## Discussion

Within the *Caenorhabditis* genus we observed significant lineage specific expansions within the NCLX exchanger group, suggesting the possibility of relatively recent gene duplication events. Within the five *Caenorhabditis* species we examined, the NCLX-type genes *ncx-6* and *ncx-7* are positioned in tandem sequence within their respective physical maps; the *ncx-8* and *ncx-9* NCLX-type genes are also in tandem sequence in the *C. elegans* genome, and *ncx-10* is closely linked on the same arm of chromosome V in *C. elegans*; in *C. briggsae ncx-8, ncx-9*, and *ncx-10* genes are all located within 15 kb of each other on chromosome V; in *C. brenneri* and *C. remanei*, *ncx-9* and *ncx-10* are within 10 kb and 3 kb of each other respectively. This linkage organization for subsets of NCLX-type exchangers may lend support to the hypothesis that some of these genes have arose relatively recently within *Caenorhabditis* species. While these apparent serial and parallel gene duplication events may be relatively recent in terms of nematode evolution, data from our group suggests that at least in the case of *ncx-6* and *ncx-9*, these genes are contributing to important neuronal functions at the behavioral and developmental levels (Vishal Sharma, Katrin Bode, and D.O'H, unpublished data), suggesting that these duplicated genes have acquired neo-functionalized roles in the animal. It will be interesting moving forward to characterize mutants in exchangers of the other NCLX-type genes in an effort to understand how sequence specificity may lend itself to functional specializations within this Na^+^/Ca^2+^ exchanger subtype. Furthermore, searching more nematode genomes as they become more annotated will add more resolution to the timing of gene accretion within the NCLX subtype by also testing the alternative possibility that these NCLX-type duplicates may have been lost in other nematode lineages. It was also surprising that we did not detect NCLX-type orthologs within the genomes of the Clade III nematodes (*B. malayi, L. loa*, and *A. suum*) that we examined. Gene loss is one possibility to explain this observation, however, it is unexpected considering the central role NCLX proteins have been shown to play in mammalian systems [18], [19], [62]. Alternative hypotheses include, diversification or low sequence coverage, each of these scenarios may have precluded their detection using our approach, and further annotation and functional analysis will be required to resolve these questions. One place that we might find clues as to the function of Na^+^/Ca^2+^ exchanger duplicates is in the case of NCX4 [59]: NCX4 has been found exclusively in teleost, amphibian, and reptilian genomes and is not present in mammalian genomes, and interestingly NCX4 is thought to have been lost from the mammalian genome [27], [59]. NCX4 has been shown to function as an NCX-type exchanger, and in zebrafish is ubiquitously expressed with highest levels in the brain and eyes [60], [61]. Reduction of NCX4 activity by morpholinos in zebrafish embryos has been shown to affect left-right patterning causing heterotaxia, situs inversus, as well as reversed cardiac looping [61]. These data demonstrate that functional specializations within the NCX family can vary significantly across species.

Na^+^/Ca^2+^ exchangers are central regulators of calcium homeostasis in a wide variety of cell types. Not surprisingly, defects in Na^+^/Ca^2+^ exchange have been implicated in numerous diseases and pathologies including epilepsy, multiple sclerosis, Parkinson's disease, Alzheimer's disease, as well as brain ischemia [21], [63]–[66]. Understanding this family of proteins means also understanding differences within this family, and an entry point into this problem is using comparative genomics to resolve structural and taxonomic specializations. This is the approach we adopted here by identifying Na^+^/Ca^2+^ exchanger genes from a broad spectrum of nematodes, and using this sequence data to reconstruct molecular phylogenetic relationships and tease apart the selective pressures shaping this family of proteins. From our analyses we uncover a pervasive theme of constraint across the Na^+^/Ca^2+^ exchanger family and reveal a significant level of heterogeneity within subtypes of this family. Specifically, in the case of the NCLX subtype of Na^+^/Ca^2+^ exchangers we observed lineage specific expansions as well as possible gene loss. Together, these findings reveal a complex picture of Na^+^/Ca^2+^ transporters in nematodes that suggests an incongruent evolutionary history of an important family of proteins that provide central control of calcium dynamics.
